# The relative contribution of causal factors in the transition from infection to clinical chlamydial disease

**DOI:** 10.1038/s41598-018-27253-z

**Published:** 2018-06-11

**Authors:** Bonnie L. Quigley, Scott Carver, Jon Hanger, Miranda E. Vidgen, Peter Timms

**Affiliations:** 1 Faculty of Science, Health, Education and Engineering, University of the Sunshine Coast, 90 Sippy Downs Drive, Sippy Downs, Queensland, 4556 Australia; 2 School of Natural Sciences, University of Tasmania, Private Bag 55, Hobart, Tasmania 7001 Australia; 3 Endeavour Veterinary Ecology, 1695 Pumicestone Road, Toorbul, Queensland, 4510 Australia

## Abstract

*Chlamydia* is a major bacterial pathogen in humans and animals globally. Yet 80% of infections never progress to clinical disease. Decades of research have generated an interconnected network linking pathogen, host, and environmental factors to disease expression, but the relative importance of these and whether they account for disease progression remains unknown. To address this, we used structural equation modeling to evaluate putative factors likely to contribute to urogenital and ocular chlamydial disease in the koala (*Phascolarctos cinereus*). These factors include *Chlamydia* detection, load, and ompA genotype; urogenital and ocular microbiomes; host sex, age, weight, body condition; breading season, time of year; location; retrovirus co-infection; and major histocompatibility complex class II (MHCII) alleles. We show different microbiological processes underpin disease progression at urogenital and ocular sites. From each category of factors, urogenital disease was most strongly predicted by chlamydial PCR detection and load, koala body condition and environmental location. In contrast, ocular disease was most strongly predicted by phylum-level Chlamydiae microbiome proportions, sampling during breeding season and co-infection with koala retrovirus subtype B. Host MHCII alleles also contributed predictive power to both disease models. Our results also show considerable uncertainty remains, suggesting major causal mechanisms are yet to be discovered.

## Introduction

*Chlamydia* is a major bacterial pathogen world-wide, affecting humans, wildlife and livestock^1–3^. Although pathologic outcomes vary between hosts and the chlamydial species involved, common clinical manifestations include urogenital and ocular inflammation that can lead to severe pain and infertility, and blindness, respectively. Other clinical conditions may occur, such as abortion, polyarthritis and pneumonia^1,4^. It is well-established that infection with *Chlamydia* does not always progress to clinical disease, with as many as 85–90% of men and women asymptomatically infected with *Chlamydia trachomatis*^5–7^. Similarly, a study of one population of koalas (*Phascolarctos cinereus*) in southeast Queensland, Australia found *Chlamydia pecorum* infection appears subclinical 70% of the time^8^. This demonstrates that chlamydial infection alone is not sufficient to predict progression to a clinical disease state. In addition, there are many well-documented cases where severe chlamydial disease pathology is observed but no chlamydial organisms can be detected, in both humans^9^ and koalas^8,10^.

Clearly, chlamydial disease progression is the result of multiple factors acting through complex mechanisms. Over the years, different studies have identified possible factors that may contribute to disease progression. Associations between the amount of chlamydial organism detected (infection load) and clinical disease presentation/severity have been made in both human^11^ and koala^12^ contexts. As well, the chlamydial serotype or genotype present (based on the Major Outer Membrane Protein gene, ompA) has been linked to the progression of symptomatic disease in humans^13^ and koalas^14^, respectively. Other organisms in the host microbiome at the infection site, especially the presence of diverse microbial populations found in conditions like bacterial vaginosis, are implicated in chlamydial infections in humans^13,15^. In the same context, co-infections with other sexually transmitted infections are also believed to have an impact^13^. At the level of host, physical characteristics like a host’s sex and age have been linked with some clinical disease presentations^14,16^, while in both humans and koalas, host immune system genetics, such as Human Leukocyte Antigen (HLA) or Major Histocompatibility Complex (MHC) gene alleles have been linked to clinical disease outcomes^17–21^. Finally, external environmental factors, such as the time of year^16^ and environmental stress from urban and agriculture development^22^ have also been associated with koalas developing chlamydial disease in the wild.

Clearly, chlamydial disease has been extensively studied in both humans and koalas, with these hosts sharing a high degree of overlap in contributing factors to disease progression. These bodies of research have revealed that humans and koalas share comparable urogenital and ocular disease presentations and outcomes^1,3^. This creates opportunities for advances in koala chlamydial disease understanding to not only benefit this vulnerable Australian marsupial, but also contribute valuable knowledge to chlamydial research in humans and other hosts.

A range of modelling studies have investigated chlamydial infection leading to disease (particularly focused on pelvic inflammatory disease^23^), but none has investigated the range of contributing factors in a unified analysis. To close this gap, we developed structural equation models (SEMs) for urogenital and ocular chlamydial clinical disease, using the koala as a model system. SEMs are used to analyse both direct and indirect relationships in a system where *a priori* knowledge of relationships between factors is available^24^. SEMs have been popular in the social and behavioral sciences for many years, and their ability to handle multi-equation models, multiple measures of concepts, and measurement error is making them applicable to a wider range of disciplines^25^. As such, SEMs can be a valuable tool to understanding how multiple factors come together in a disease setting. Using this approach, we determined that different contributing factors lead to clinical urogenital and ocular chlamydial disease. We also show that considerable unexplained variation remains and, thus, more factors may need to be considered in order to understand the progression of chlamydial infection to clinical disease.

## Results

### Establishing the urogenital and ocular disease models

#### Evaluating factors for the structural equation models

To model what factors predict clinical urogenital and ocular chlamydial disease, data from 204 wild koalas were collected on a range of chlamydial infection, environmental, koala physical and microbiome, and co-infection parameters (Table 1, extended descriptions in Supplementary Table 1 and Supplementary Methods). Ocular microbiome data was only obtainable from 111 koalas, hence the ocular clinical disease model was limited to the 111 koalas for which all data were available.

**Table 1:** Summary of chlamydial disease factors considered for disease modelling.

Category	Factor	Description of factor	Alternative versions of factor (if considered)
Clinical disease status	Urogenital disease	Clinical reproductive and/or urinary/renal disease as assessed by physical exam and ultrasound	
	Ocular disease	Clinical ocular disease as assessed by physical exam and ultrasound	
	Any chlamydial disease	Clinical reproductive, urinary/renal and/or ocular disease as assessed by physical exam and ultrasound	
Host physical	Age	Koala age assessed by tooth wear or known interaction history	
	Sex	Koala sex as assessed by physical exam	
	Weight	Koala weight (in kg)	
	Body condition	Koala overall body condition, assessed by physical exam on 6 point scale; 3 (poor) - 9 (excellent)	
Environmental	Time of year	Time of year sample collected	4 seasons (spring/summer/autumn/winter); breeding season (out [Jan-Aug]/in [Sept-Dec]); breeding season longer (out [Feb-Jun]/in [Jul-Jan])
	Location in study site	Subsite location (1–5) within study region	
*Chlamydia* infection	Infection status	Detection of *Chlamydia pecorum* at site via qPCR	2-level detection (no/yes); 3-level detection (no/low positive (<100 copies/µl)/high positive (>100 copies/µl))
	Infection load	qPCR copies/µl sample detected	copies/sample; log transformed copies/sample
	*Chlamydia* genotype	ompA genotype (E’, F, G, mixed)	
Co-infections	Koala retrovirus detection	KoRV-B provirus detected in host genome via conventional PCR	
	Koala retrovirus subtype	Sequence type based on envelope protein fragment	
Microbiome	Overall composition	Percentage of each sample microbiome composed of group indicated	Phylum-level; genus-level; OTU-level
	Single OTU dominated	Microbiome comprised of >75% single OTU (monolithic)	
	Cluster analysis	Grouping of OTU-level microbiome composition by 75% Bray-Curtis dissimilarity values	
Host MHC class II genetics	Gene class profiles	DAb allele profile; DBb allele profile	
	Individual alleles	DAb*10; DAb*15; DAb*19; DAb*21; DAb*30; DAb*31; DAb*32; DAb*33; DAb*34; DAb*35; DAb*36; DBb*01; DBb*02; DBb*03; DBb*05	

**Figure 1:**
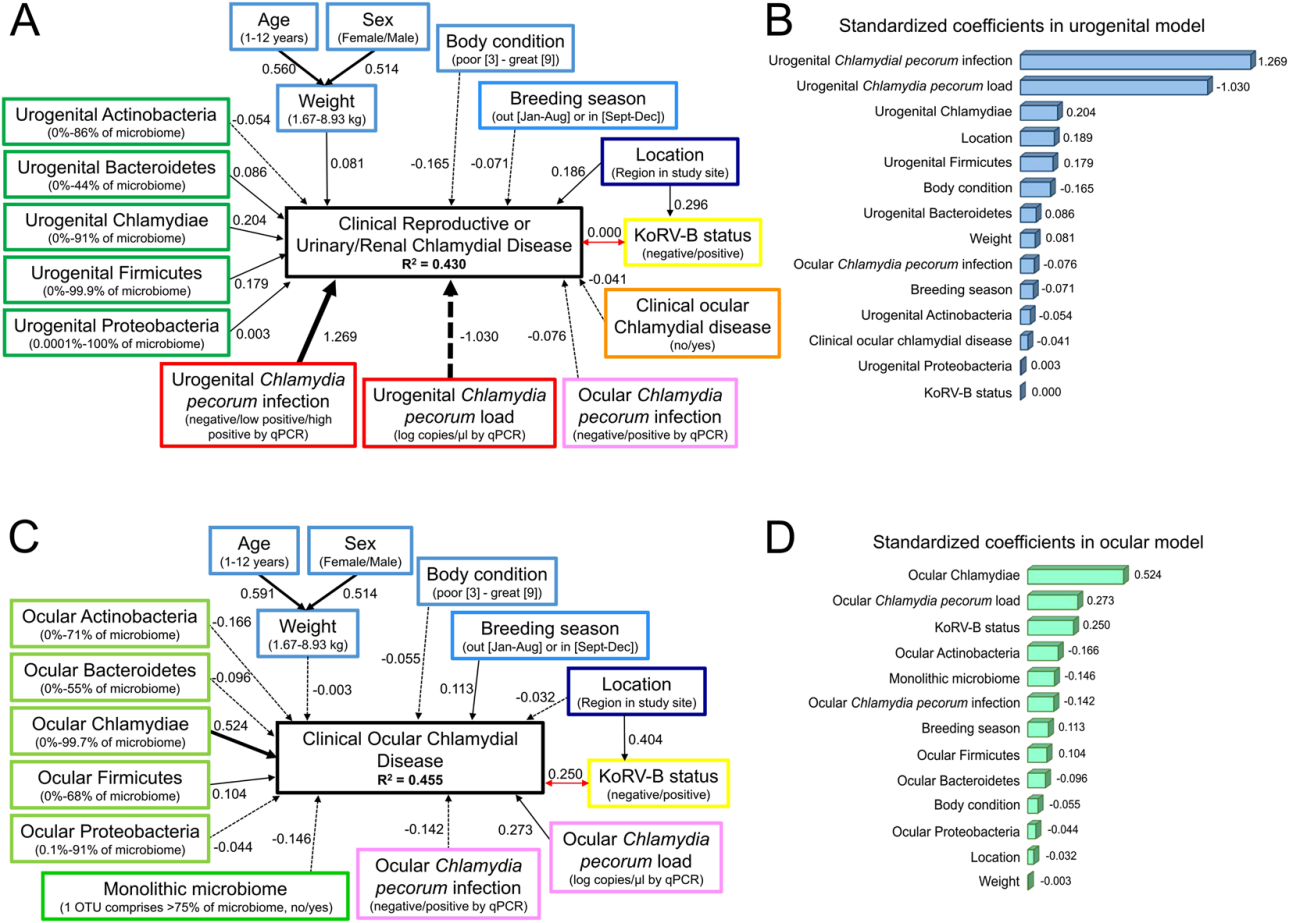
Structural equation models diagramming the factors influencing chlamydial disease at (**A**) urogenital (n = 204) or (**C**) ocular (n = 111) sites. Factors considered in the model are diagrammed in boxes with descriptions of that factor in parenthesis. The value on the arrow represents the amount of variance explained by the factor at the start of the arrow on the factor at the end of the arrow. The farther the value is from zero, the larger the influence (thicker the arrow in the diagram), with values comparable across the model. Solid arrows indicate positive effect; dashed arrows indicate negative effect; double-headed red arrows indicate co-variance. Summary of the standardized coefficients from (**B**) urogenital and (**D**) ocular models.

Preliminary structural equation models were designed as starting models based on *a priori* knowledge of urogenital and ocular chlamydial disease in koalas. Then, through a systematic backward elimination process with model selection, using Akaike Information Criterion corrected for small sample size (AICc), the most parsimonious models describing chlamydial disease status was determined. Each model alternative had to satisfy basic model fit parameters to be considered valid, then AICc was used to assess the relative importance of models, and R^2^ goodness of fit. If the model version being tested generated a lower AICc than the starting model, the test model became the new starting model for further testing (Supplementary Table 2). A final model for urogenital and ocular disease was reached when backward elimination no longer improved AICc. SEM analysis revealed that, based on the factors that best modelled the data, 43.0% of clinical urogenital disease (Fig. 1A) and 45.5% of clinical ocular disease (Fig. 1C) could be accounted for.

#### Factors included and excluded from the final models

**Figure 2:**
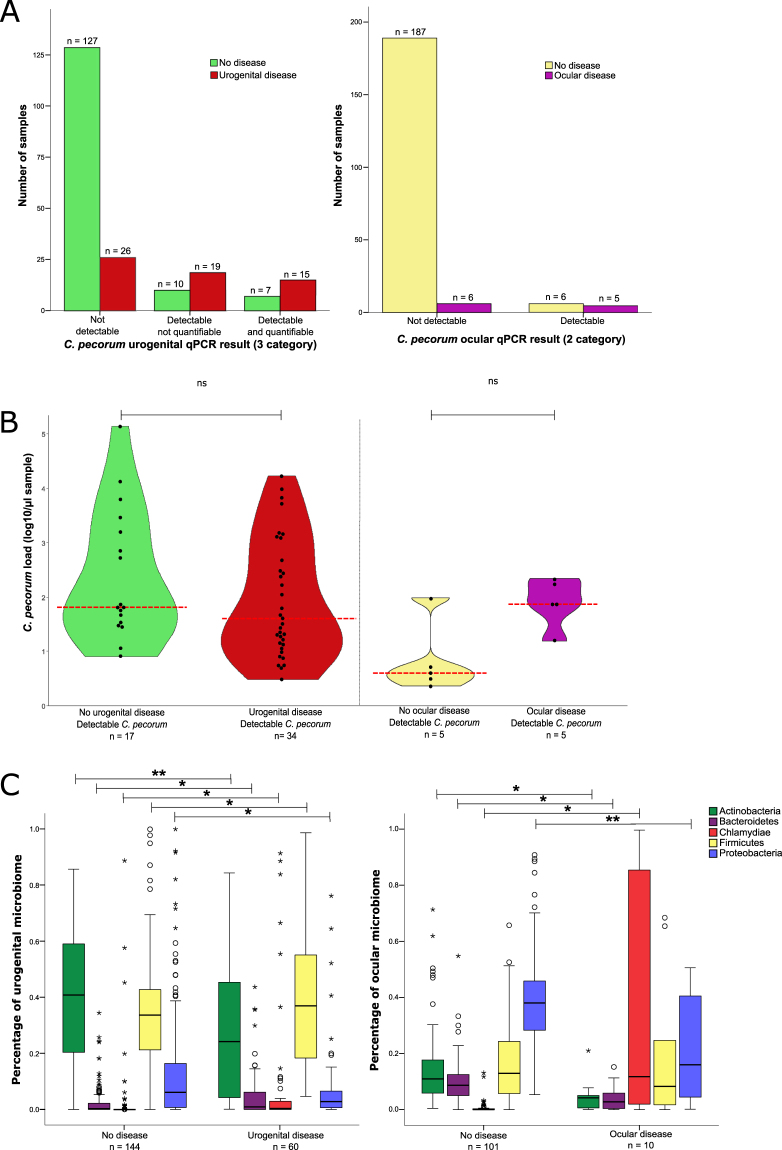
Microbiological factors that influence *Chlamydia* disease modelling. (**A**) *C*. *pecorum* detection via qPCR, at either 3 levels of detection for the urogenital site (not detectable, detectable not quantifiable (<100 copies/µl sample) and detectable and quantifiable (>100 copies/µl sample) or 2 levels of detection for the ocular site (not detectable and detectable). (**B**) Non-zero copy number of *C*. *pecorum* 16 S rRNA genes detected in urogenital and ocular samples via qPCR by clinical disease states. Median values are indicated by the red dashed line. (**C**) Microbiome compositions at the phylum level of urogenital and ocular sites by clinical disease state. Significances are indicated by *(p < 0.05), **(p < 0.01) or not significantly different (ns).

From the factors related to *C*. *pecorum*, both models retained a measure of infection status (detection of *C*. *pecorum* at the site by qPCR). A 3-level *C*. *pecorum* detection factor (not detected, detected at low levels (below 100 copies/µl of sample), and detected at high levels (above 100 copies/µl of sample)) was more optimal for the prediction of urogenital disease while a 2-level *C*. *pecorum* detection factor (not detected verses detected) was preferred in the ocular model (Fig. 2A**)**. Both urogenital and ocular models retained a factor for *C*. *pecorum* load (in log copies/µl of sample) (Fig. 2B) while neither model found *C*. *pecorum* ompA genotype improved the model (therefore, *C*. *pecorum* genotype was removed from both models).

Since microbiome composition could be represented at a range of levels, factors were created that summarized these data at the phylum-level (for the five major phyla detected, Fig. 2C), the genus-level (for genera that had statistically different means between healthy and clinical disease states, Supplementary Fig. 1) and OTU-level (manually chosen from Bray-Curtis dissimilarity clustering analysis, Supplementary Figs 2 and 3). Surprisingly, the phylum-level data generated the best AICc statistics in model evaluation as the preferred format of the factor. An interesting observation about the phylum-level Chlamydiae group was that it comprised of at least 99.9% *C*. *pecorum* in all samples. All non-*C*. *pecorum* Chlamydiae were detected in both urogenital and ocular microbiomes made up no more than 0.1% of the total phylum-level Chlamydiae^26^. Non-taxonomic grouping strategies, such as clustering analysis, generated poorer AICc statistics than phylum-based grouping (Supplementary Figs 2 and 3 and Supplementary Table 2). Finally, a factor was created to indicate whether the microbiome was dominated (comprised of >75%) by a single OTU. This “monolithic microbiome” factor improved the model for ocular clinical disease only and was retained there.

**Figure 3:**
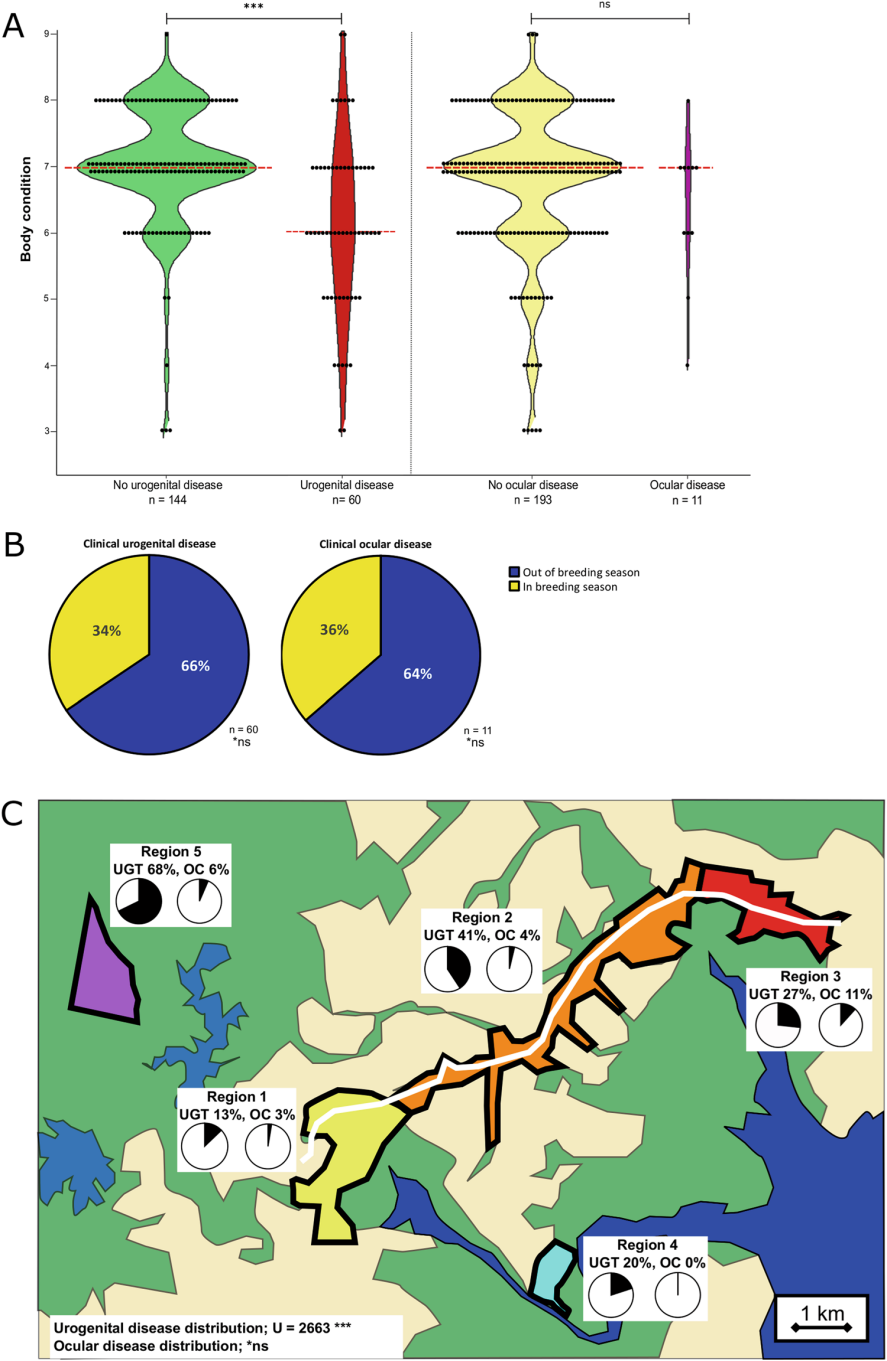
Physical and environmental factors that influence *Chlamydia* disease modelling. (**A**) Body conditions by urogenital and ocular disease. Median values are indicated by the red dashed line. (**B**) Breakdown of clinical urogenital and ocular disease observed by breeding season. (**C**) Distribution of urogenital (UGT) and ocular (OC) disease within the regions of the study site. Background colours on the map represent water (blue), green space (green) and urban development (tan). The white line represents a train line through the area. Pie charts represent chlamydial disease within a region with black slices indicating disease and white slices indicating health. Significances are indicated by ***(p < 0.001), and not significantly different (*ns).

All the koala physical factors considered (sex, age, weight and overall body condition (Fig. 3A)) were mathematically advantageous to both models and were retained in the final models. From the environmental factors evaluated, sampling for disease in the context of a short koala breeding season (September to December) (Fig. 3B) improved both disease models compared to a longer breeding season (July to January) and seasons of the year (spring/summer/autumn/winter). Location within the study site, which served as a proxy to capture environmental differences such as food availability, disturbance by urban development, predators in the area, and existing pockets of chlamydial disease (Fig. 3C), was a factor that improved both urogenital and ocular models.

**Figure 4:**
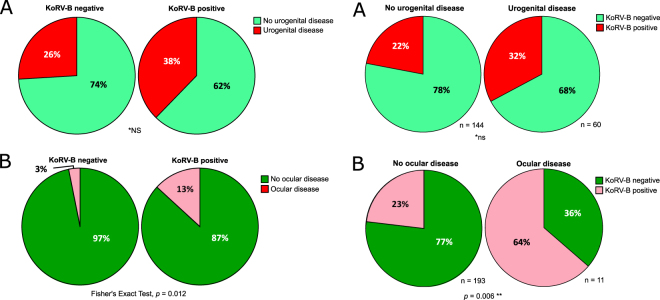
Co-infection status with koala retrovirus subtype B (KoRV-B) in (**A**) urogenital and (**B**) ocular clinical disease. Significances are indicated by ** (p < 0.01) and not significantly different *(ns).

Finally, evaluation of co-infection was done by considering both koala retrovirus (KoRV) and chlamydial disease at the body site not being actively modeled. KoRV was captured with a factor for KoRV-B infection (all koalas were KoRV-A positive) (Fig. 4). KoRV-B infection was included as a co-variance with clinical chlamydial disease, since it is currently unknown how KoRV infection affects chlamydial disease and vice versa. A factor representing the different envelope types of KoRV-B in the population was tested in each model and found not to enhance either model. Chlamydial disease at the alternative body site was evaluated as both an infection factor (*C*. *pecorum* not detected/detected by qPCR) and clinical disease assessed at the site. For urogenital disease, the ocular infection and disease factors improved the model and were retained. For ocular disease, the urogenital infection and disease factors did not improve the model and were removed.

### Urogenital and ocular disease progression is influenced by different factors

#### Microbiological influences on urogenital and ocular disease

For urogenital disease prediction, the detection (standardised coefficient (SC) = 1.269) and load (SC = −1.030) of *C*. *pecorum* at the infection site were by far the most predictive disease factors (Fig. 1A,B). The presence or absence of detectable *C*. *pecorum* corresponded to the clinical disease status in 80% of cases (Fig. 2A). *C*. *pecorum* load contribute to disease prediction with non-zero detectable loads found in 57% of disease cases. The negative effect of load resulted from a slightly lower median level of *C*. *pecorum* detected in clinically diseased koalas (44 copies/µl) compared to infected, asymptomatic koalas (65 copies/µl) (Fig. 2B). However, this load difference was non-significant (t(49) = 1.255, p = 0.215) and only 11% of healthy koalas carried *C*. *pecorum* asymptomatically while 57% of clinically diseased koalas were infected. Additionally, microbiome phylum-level factors contributed approximately five times less than direct *C*. *pecorum* factors, with increasing levels of Firmicutes (SC = 0.179) and Chlamydiae (SC = 0.204) detected in the urogenital microbiome adding to disease prediction (Fig. 2C). Together microbiological methods reveal that 82% of clinical urogenital disease cases and only 31% of asymptomatic infected koalas had detectable *C*. *pecorum* (or a close relative) (Table 2).

**Table 2:** Summary of *Chlamydial* microbiological contributors to disease prediction.

Disease site	Disease status	Number of koalas				
		*C*. *pecorum* detectable by qPCR	*C*. *pecorum* detected in microbiome	Any non-*C*. *pecorum* Chlamydiae detected in microbiome	Any Chlamydiae (including *C*. *pecorum*) detected in microbiome	No *C*. *pecorum* detected by qPCR AND No other *Chlamydiae* detected in microbiome
Urogenital	Disease (n = 60)	34 (57%)	48 (80%)	2 (3%)	49 (82%)	11 (18%)
	Healthy (n = 144)	17 (12%)	40 (28%)	7 (5%)	45 (31%)	99 (69%)
Ocular	Disease (n = 11)	5 (45%)	9 (82%)	2 (18%)	9 (82%)	2 (18%)
	Healthy (n = 193)	5 (3%)	na	na	na	na
	Healthy with microbiome data (n = 101)	5 (5%)	36 (36%)	11 (11%)	43 (43%)	58 (57%)

Conversely, detection (SC = −0.142) and load (SC = 0.273) of *C*. *pecorum* was much less predictive for ocular disease prediction (Fig. 1C,D). Detection of *C*. *pecorum* corresponded with active clinical disease in only 45% of cases (Fig. 2A) and there was no significant difference between *C*. *pecorum* non-zero loads in infected, asymptomatic koalas (median 4 copies/µl, 3% of ocular healthy koalas) and ocular diseased koalas (median 76 copies/µl, 55% of diseased koalas); t(9) = −2.059, p = 0.070. In ocular disease, the most predictive factor was the proportion of the ocular microbiome that was comprised of Chlamydiae (SC = 0.524). Koalas with clinical ocular disease had detectable Chlamydiae in their eye in 82% of cases (Table 2). Within these diseased Chlamydiae-positive koalas, the bacteria ranged from 1–92% of the total ocular microbiome. This contrasted with ocular healthy koalas, which had detectable Chlamydiae in their eye in only 43% of cases, with ranges of <0.1–12% of the total ocular microbiome. Also unique to the ocular disease analysis, it was determined that having a diverse microbiome contributed predictive power to clinical ocular disease (SC = −0.146).

#### Physical influences on urogenital and ocular disease

In both urogenital and ocular disease models, respectively, both age (SC = 0.560 and 0.591) and sex (SC = 0.514 and 0.514) strongly predicted a koala’s weight, but weight was not a major predictor of either clinical disease status (SC = 0.081 and −0.003) (Fig. 1A,C). A koala’s body condition was much more predictive for urogenital disease (SC = −0.165) than ocular disease (SC = −0.055), suggesting that koalas with poor body condition (for whatever reason – such as fighting, injury, other illnesses, or current chlamydial disease) were more likely to be diagnosed with urogenital disease compared to ocular disease. This was reflected in the population where koalas with clinical urogenital disease had a median body score of 6 compared to urogenital healthy koalas’ median body score of 7 (Mann-Whitney U = 2511, p < 0.001). Ocular diseased and healthy koalas both had median body scores of 7 (Mann-Whitney U = 833, p = 0.200) (Fig. 3A).

#### Environmental influences on urogenital and ocular disease

For urogenital disease prediction, the time of year the koala was sampled (in or out of their breeding season) had virtually no predictive power on clinical disease presentation (SC = −0.071) (Fig. 1A). Conversely, for ocular disease, there was some positive predictive power of sampling during the breeding season (SC = 0.113) (Fig. 1C). Based on the time of year when koalas were diagnosed with clinical disease, 34% of urogenital disease and 36% of ocular disease was seen during breeding season (non-significant proportions) (Fig. 3B). The location where the koala was found in the study site had some predictive power for urogenital disease (SC = 0.186), but not for ocular disease (SC = −0.032). This is in agreement with the known distribution of urogenital and ocular disease in this area, with urogenital disease distribution significantly localized to some areas (Mann-Whitney U = 2663, p < 0.001) while ocular disease had a uniform distribution in the study site (non-significant distribution) (Fig. 3C).

#### Co-infection

Koalas with clinical urogenital disease were just as likely to be infected with KoRV-B (32%) as koalas without urogenital disease (22%) (χ^2^(1) = 2.015, p = 0.156), resulting in this factor having no predictive power (SC = 0.000) (Figs 1A and 4A). By comparison, koalas with clinical ocular disease were significantly more likely to be infected with KoRV-B (64%) than koalas without ocular disease (23%) (Fisher’s Exact Test, *p* = 0.006), lending much more predictive power to this factor (SC = 0.250) (Figs 1B and 4B).

While the inclusion of ocular infection and disease factors added to the overall model fit for urogenital disease, the analysis revealed that knowing the ocular chlamydial infection and disease status contributed very little to urogenital disease prediction (SC = −0.076 and −0.041, respectively).

### Major factors still missing from clinical disease explanations

#### Major Histocompatibility Complex (MHC) host genetics

Given that the current models only account for 43% of urogenital disease and 46% of ocular disease, it was clear that addition factors are important for clinical disease progression. As a preliminary next step, we identified a set of MHC class II gene alleles for a subset of the koalas in this study (DAb and DBb gene alleles, n = 57). From our koalas, 11 alleles of DAb were detected, of which five have been previously reported (DAb*10, DAb*15, DAb*19, DAb*21, and DAb*30) and six are novel to this study (DAb*31 to DAb*36). All four of the DBb alleles detected in this population have been previously reported (DBb*01, DBb*02, DBb*03, and DBb*05) (Supplementary Fig. 4). Evaluated individually, DBb*03 was the only allele to be associated with a clinical outcome; overall clinical chlamydial disease (at any body site) was associated with the absence of DBb*03 (χ^2^(1) = 4.466, *p* = 0.035).

We took our best fit ocular and urogenital SEMs and explored if adding MHC factors added greater explanatory power to chlamydial disease status. This is necessarily a preliminary analysis, as reducing the number of koalas for model generation, as well as adding additional free parameters, challenges standard rules for the number of samples/factor recommended in SEMs^27–29^. MHC alleles were tested by adding each allele factor to the existing model either individually, or as a composite DAb or DBb profile factor. Factors that improved model goodness of fit were combined into a final preliminary MHC-inclusive model for each body site. In both urogenital and ocular models, our tentative findings indicate that the addition of alleles DAb*21, and DAb*32 to DAb*36 added to the variation in chlamydial disease explained, with an additional 7% of urogenital disease and 2% of ocular disease accounted for with this additional host immune genetic information (Supplementary Figs 5 and 6).

## Discussion

Over the many years of *Chlamydia* research, with multiple hosts and species of *Chlamydia* pathogens, a disconnect between infection and disease has existed: infection with a chlamydial pathogen does not always lead to clinical disease and, during clinical disease, the instigating chlamydial pathogen cannot always be detected. This suggests that more than just the chlamydial pathogen is necessary to progress from infection to disease and that clinical signs and pathological changes can persist with undetectable levels of the chlamydial organism. Over the years, several microbiological, host and environmental factors have been implicated in contributing to clinical disease, but no attempt had yet been made to combine these factors into a predictive model of clinical chlamydial disease to aid in understanding their complex relationships. To redress this gap, this study unified a range of factors that were known or hypothesized to contribute to clinical chlamydial disease in the koala, and used SEM to model their effect on chlamydial disease prediction. Although koalas were used as a model in this analysis, many of the same factors have been implicated in chlamydial disease in humans and other animals. Therefore, advances in our understanding of chlamydial disease factors in general has implications that reach across host species and health/medical disciplines. The parallels between koala *C*. *pecorum* and human *C*. *trachomatis* disease, in particular, make findings in koalas very relevant to human *Chlamydia* research. The result from our analysis has been a clear separation of chlamydial urogenital and ocular diseases as separate processes, governed by distinct microbiological, host and environmental factors. In addition, despite the breadth of factors already under active research consideration, more than half of the clinical disease observed at each body site is influenced by factors that are not commonly considered.

Not surprisingly, the most heavily weighted factors that influenced disease progression in these models were the *C*. *pecorum* microbiological factors. What was interesting was the finding that *C*. *pecorum* results (detection and load) were better predictors of urogenital disease while phylum-level Chlamydiae microbiome proportions were better at predicting ocular disease. The poor association of chlamydial load with ocular disease noted here is consistent with immunohistochemistry studies, where inclusions can be rare despite florid conjunctival inflammation and tissue proliferation^9,30^. Within the context of a multi-factor regression model, the ability of the qPCR to perform better at detecting *C*. *pecorum* in urogenital disease situations added to its significance while the larger difference in *C*. *pecorum* microbiome proportions between ocular disease states gave that factor more weight in the ocular disease model. These subtle differences, highlighted by the SEM approach, reflect genuine biological differences between *C*. *pecorum* infections at the two body sites.

The other factors considered in these disease models contributed relatively smaller proportions to the overall predictive power of clinical chlamydial disease, however, they continued to highlight the differences between urogenital and ocular disease. A koala’s body condition and environmental location had the greatest impacts on whether an animal was predicted to have urogenital disease. The way the body condition score was incorporated in the models meant that if a koala was captured from the wild and scored with a lower overall body score, it was more likely to have urogenital disease than ocular disease. This could represent either a situation where a koala was impacted by an injury or unrelated illness that predisposed them to urogenital chlamydial disease development or that a koala had urogenital disease prior to its capture and that disease state had more of an impact on its body condition than ocular disease would. It is, of course, accepted that major injury or prolonged chlamydial disease at either site would negatively impact body condition, but in the context of the factors considered in each model, body condition decline was more predictive of urogenital disease than ocular disease. Additionally, the environmental location of the koala had an impact on whether it was predicted to have active urogenital disease. Different study locations had different levels of stressors, which included more interaction with urban housing, different food availability, and different predators in the area. Interestingly, the region with the highest urogenital chlamydial disease (region 5, 68%) was a greenspace away from the urban housing developments. This implies that close contact with human habitation is not a sole indicator of an environment where koalas are more likely to develop chlamydial disease. How interactions with humans, predators, space and food resources influence chlamydial disease progression is currently not understood, but these results show that the location impacts urogenital disease differently than ocular disease.

From the ocular disease perspective, sampling during breeding season and co-infection with KoRV-B were the non-microbiological factors that contributed most to clinical disease prediction. The link to breeding season suggests that contact between koalas during mating or male-to-male fighting over territory or mates may contribute to this factor’s predictive power for ocular disease during this time. It is also possible that stressors and/or hormones during mating season influence ocular disease signs differently than urogenital signs. Another contributor was co-infection with KoRV-B. Infection with KoRV-B has been linked to clinical chlamydial disease^8,31^. Within these models, co-infection with KoRV-B was significantly linked to more clinical ocular disease only. In previous KoRV-B/*Chlamydia* studies, as well as in our current models, the severity of urogenital and ocular disease was not factored into the analysis, nor was the type of urogenital disease (urinary tract disease verses reproductive tract disease). Defining both urogenital and ocular disease more precisely in future analyses may help focus the results of KoRV-B influence onto the key chlamydial disease conditions affected. At present, the mechanism of interaction between KoRV and chlamydial disease is currently unknown and is an area of active research.

Beyond the factors considered in these chlamydial disease models, this analysis clearly demonstrated considerable unexplained variation in determining the progression to both urogenital and ocular disease. In their current form, the models do not contain any *Chlamydia* cellular properties, host genetic components or immune response measures. It was surprising that chlamydial genotype (based on the ompA surface protein) did not improve the models, as this factor has been linked to clinical disease in the past^14^. However, other chlamydial properties, such as the presence of plasmids^32^, type III secretion system properties^33^, activities of self-peptide presentation mechanisms^34^, or the ability to down regulate MHC class I molecules^35^, are all alternative characteristics that could add predictive power to a clinical disease model. From the host genetic component, preliminary analysis suggested that host MHC class II gene alleles do contribute predictive power to clinical chlamydial disease. Additional host genes that could be considered further include interleukin (IL)−12B, IL-10, tumor necrosis factor (TNF)-α, and interferon (IFN)-γ genes^17,36,37^. Finally, measuring the immune response directly will undoubtedly contribute to the modeling of disease progression. IFN-γ levels (often considered the primary driver of a protective chlamydial host response and optimal antibody-mediated immunity)^37,38^, T-cell responses^39^ and B-cell responses^40^ would all be valuable factors to investigate in future modeling. Despite the absence of these factors in the current analysis, an advantage of the approach taken in this study is that the models generated give an indication of the relative importance of both the factors considered and of the proportion of “missing” data yet to be investigated. Importantly, this research suggests major causal mechanisms of chlamydial disease are yet to be discovered.

There are many parallels in symptomatic chlamydial disease between hosts. Many of the factors that have been shown or hypothesized as important in koala *Chlamydia* pathogenesis have counterparts in human *Chlamydia* research. The chlamydial SEM models generated in this study demonstrate that modeling can successfully find associations and relationships that might otherwise be missed in individual, factor-focused studies. Additionally, by looking at several factors in a combined approach, the relative weight and importance of each factor can be assessed in the context of all the others. This makes SEM a powerful tool to unravel the complexity of chlamydial pathogenesis in any species.

## Materials and Methods

### Animals

Koalas included in the study (n = 204) were part of a multi-year population-wide management program by the Queensland Government Department of Transport and Main Roads for the Moreton Bay Rail (MBR) project, in the Moreton Bay Region, Queensland, Australia (project centre-point: 27.25°S,153.02°E). All procedures were approved by the University of the Sunshine Coast (USC) Animal Ethics Committee (Animal ethics number AN/A/13/80) and by the Queensland Government (Scientific Purposes Permit, WISP11532912). All experiments were performed in accordance with relevant guidelines and regulations.Detailed description of koala sampling has been previously reported^8^ and is found in Supplementary Methods.

### Model parameter data

Data used for clinical disease parameters, physical koala characteristics, location parameters, *Chlamydia* infection parameters and KoRV infection parameters have all been previously reported^8^. The urogenital and ocular microbiome data referred to in this study were generated previously^26^. Detailed descriptions of parameter data is given in Supplementary Methods.

### Host genetics

For a subset of koalas (n = 57), MHC class II DAb and DBb gene alleles were determined. Koalas were selected from the study population by determining which animals had the greatest depth in their ocular microbiome dataset (as the ocular microbiome data parameter was the experimental parameter with the least overall sequencing depth available). The 57 koalas with the most ocular microbiome reads maintained a comparable representation of chlamydia disease states and KoRV-B results to the overall population and were deemed suitable as a subset for MHC examination. Using the DNA extracted from whole blood, PCR for the DAb (271 bp product) and DBb (282 bp product) gene was carried out as previously described^21,41^. PCR products were cloned into pGEM-T-Easy TA cloning vector system (Promega) as per manufacturer’s instructions. Six clones per gene target per sample were isolated and sequenced (Macrogen, Inc). Sequences were examined to determine the number and allele type present in each animal. Novel allele types (DAb*31 to DAB*36) have been deposited in Genbank under the accession numbers MG957484-MG957489.

### Structural equation modeling

Preliminary structural equation models were designed as starting models based on *a priori* knowledge of urogenital and ocular chlamydial disease in koalas (Supplementary Fig. 7). Then, using the lavaan package of R^42^, systematic model evaluation was undertaken to test factors using a backward elimination process and model selection to find the most parsimonious model (Supplementary Table 2). Each model tested had to satisfy three model fit parameters: Bollen-Stine P-value (>0.05), the Confirmatory Fit Index (CFI) (>0.9) and the Root Mean Square Error of Approximation (RMSEA) (<0.1). Once these parameters were satisfied, the Akaike Information Criterion corrected for small-sample-size (AICc) was used to assess the relative importance of a model and R^2^ calculated to estimate the goodness of fit for the model, using the AICcmodavg package in R^43^. If the model version being tested generated a lower AICc, the test model became the new starting model for further backward elimination testing (Supplementary Table 2). A final model for urogenital and ocular disease was determined when no further variable elimination improved model fit by AICc.

### Data availability

Accession numbers for MHC class II allele sequences generated in this study can be found at GenBank MG957484-MG957489.

## Electronic supplementary material

Supplementary material
