# Supplementary material for: The relative contribution of causal factors in the transition from infection to clinical chlamydial disease
Source: Sci Rep. 2018 Jun 11;8:8893. doi: 10.1038/s41598-018-27253-z (PMC5995861; doi:10.1038/s41598-018-27253-z)
Supplement: Supplementary file 1 — Supplementary material [file 41598_2018_27253_MOESM1_ESM.pdf]

Supplementary material for:

The relative contribution of causal factors in the transition from infection to clinical chlamydial disease

Bonnie L Quigley, Scott Carver, Jon Hanger, Miranda E Vidgen, and Peter Timms

Supplementary Methods
Supplementary Table 1. Extended description of factors considered in the structural equation models
Supplementary Table 2. Structural equation modeling statistics for systematic model evaluation
Supplementary figure 1. Genera with significant differences between clinically diseased and healthy koalas at urogenital and ocular sites.
Supplementary figure 2. Urogenital microbiomes at the OTU level. OTU that comprised at least 10% of at least one microbiome (63 OTU in total) were hierarchically clustered (with average linkage) based on Bray-Curtis dissimilarity values.
Supplementary figure 3. Ocular microbiomes at the OTU level. OTU that comprised at least 10% of at least one microbiome (63 OTU in total) were hierarchically clustered (with average linkage) based on Bray-Curtis dissimilarity values.
Supplementary figure 4. Major Histocompatibility Complex (MHC) class II gene allele results from koalas (n = 57).
Supplementary figure 5. Preliminary model of clinical urogenital disease with MHC class II gene data added.
Supplementary figure 6. Preliminary model of clinical ocular disease with MHC class II gene data added.
Supplementary figure 7. Starting models for urogenital (A) and ocular (B) structural equation modeling.

Supplementary Methods

Animals. Koalas included in the study (n = 204) were part of a multi-year population-wide management program by the Queensland Government Department of Transport and Main Roads for the Moreton Bay Rail (MBR) project, in the Moreton Bay Region, Queensland, Australia (project centre-point: 27.25° S,153.02°E) . Detailed description of koala sampling has been given previously[1]. Koalas in this population were captured, clinically examined by experienced wildlife veterinarians and released back into the wild. Ocular and urogenital swabs (for *C. pecorum* testing and microbiome analysis), as well as blood samples (for KoRV testing), were collected from koalas under general anaesthesia during veterinary examinations and stored at -20°C until transport to the laboratory, where they were stored at -80°C until processing. All procedures were approved by the University of the Sunshine Coast (USC) Animal Ethics Committee (Animal ethics number AN/A/13/80) and by the Queensland Government (Scientific Purposes Permit, WISP11532912). All experiments were performed in accordance with relevant guidelines and regulations.

Physical characteristics and clinical disease parameter data. Determination of koala age (*Age*: rounded to the nearest whole year based on tooth wear or known date of birth), sex (*Sex*: male/female/intersex), weight (*Weight*: in kg), body condition (*Body_condition*: scored from 3 (poor) to 9 (great)) and chlamydial disease status (reproductive and/or urinary/renal and/or ocular, determined by veterinary examination findings) at time of sampling were documented by the examining veterinarian. Veterinary examination

consisted of a general physical examination including palpation of muscles/bones/joints/peripheral lymph nodes, examination of skin/coat condition (including parasite check), eyes, nose, oral cavity, stomach/abdomen, pouch, and reproductive organs, as well as ultrasound of the bladder, kidneys, ureters, and reproductive tract. Also collected for analysis were blood, urine, bone marrow and abdominal aspirate. For the purposes of our analysis, clinical disease was coded into three parameters: *Any_clinical_disease* (disease detected at any body site), *Vet_exam_Cp_UGT_urinary_renal* (reproductive and urinary/renal chlamydial disease were grouped together as urogenital disease) and *Vet_exam_Cp_ocular* (ocular disease only).

Environmental characteristics parameter data. The time of sampling parameters, *4Season* (spring (Sept, Oct, Nov)/summer (Dec, Jan, Feb)/autumn (Mar, Apr, May)/winter (Jun, Jul, Aug)), *Breeding_season* (breeding season (Sept-Dec), not breeding season (Jan-Aug)), and *Breeding_season_longer* (breeding season (Jul-Jan), not breeding season (Feb-Jun)) were assigned based on the koala sampling date. The parameter *Location_simple* was assigned based on the capture point of the koala within the study area on the sampling day. The study area was arbitrarily divided into 5 polygons. The *Location_simple* parameter served as a proxy for general environmental conditions in the polygon, such as food availability, potential stressors (such as people or noise) and predators, as well as any other location-specific differences.

Chlamydia infection parameters (both urogenital and ocular). The *C. pecorum* infection status of the animals used in this study has been reported previously[1]. Briefly, DNA was extracted from urogenital and ocular swab samples as previously described[2]. These were screened for the presence of *C. pecorum* using a diagnostic quantitative real-time PCR (qPCR) targeting a 204 bp fragment of the *C. pecorum* 16S rRNA gene. Assays were as described previously[3,4] and results were coded into urogenital (UGT) or ocular (OC) parameters *Cp_UGT_qPCR_results2/Cp_OC_qPCR_results2* (not detectable/detectable), *Cp_UGT_qPCR_results3/ Cp_OC_qPCR_results3* (not detectable/detectable but not quantifiable (below 100 copies/µl of sample tested)/detectable and quantifiable), *Cp_UGT_qPCR_load/ Cp_OC_qPCR_load* (actual copies/µl detected in sample tested), and *Cp_UGT_qPCR_load_log/ Cp_OC_qPCR_load_log* (log10 transformed copies/µl detected in sample tested). Samples that were positive for *C. pecorum* were further typed based on amplification and sequencing the variable 3-4 region of the ompA gene by with PCR primers (CpeNTVD3/CpeNTVD4) and conditions described previoulsy[3]. Identified ompA sequence types (no chlamydia, genotype E', genotype F, genotype G, mixed infection, sample positive but not typed) were coded into the parameters *UGT_ompA_type/OC_ompA_type*.

Co-infection status with koala retrovirus (KoRV). The KoRV infections status of the animals used in this study have been reported previously[1]. Briefly, DNA was extracted from whole blood and KoRV-B subtype specific conventional PCR (targeting a 271 bp product from the envelope gene region) was performed[1]. Detectable/not detectable

results were coded into the parameter *KoRV_B*. Samples that generated KoRV-B envelope gene fragments were sequenced and the 21 unique sequence types generated were coded into parameter *KoRV_B_epi_subtype*.

Microbiome data. The urogenital and ocular microbiome data referred to in this study were generated as part of Vidgen *et al.*[5]. Briefly, DNA extracted from urogenital and ocular swabs was amplified using standard PCR primers to the 16S rRNA gene V3-V4 region and sequenced with Illumina MiSeq technology. Data processing is extensively described previously[5], resulting in QIIME-processed OTUs that were assigned various taxonomic levels using the Silva 119 database. Koalas with microbiome read counts above 100 reads/sample were included in this analysis. Read counts for each sample were converted to relative abundance for each taxonomic level.

For phylum-level analysis, the proportion of each sample microbiome, comprised of five major phyla, was coded into the urogenital (UGT) or ocular (OC) parameters *UGT/OC_Actinobacteria, UGT/OC_Bacteroidetes, UGT/OC_Chlamydiae, UGT/OC_Firmicutes*, and *UGT/OC_Proteobacteria*.

For genus-level analysis, genera identified were filtered to included genera that were at least 5% of at least one microbiome and then assessed for significant proportional differences between no disease verses urogenital or ocular disease states. Genera that were significantly different ($p < 0.05$) by independent samples t-test (performed in SPSS (IBM Corp. Released 2013. IBM SPSS Statistics for Windows, Version 22.0. Armonk, NY: IBM Corp.)) were coded into the parameters

UGT_Aerococcus, UGT_Chlamydia, UGT_Corynebacterium, UGT_Facklamia, OC_Chlamydia and *OC_Flavobacterium* (**Supplementary figure 1**).

For OTU-level analysis, OTU that comprised at least 10% of at least one microbiome (63 OTU in total) were hierarchically clustered (with average linkage) based on Bray-Curtis dissimilarity values generated with the vegan package in R [6] (**Supplementary figure 2 and 3**). Clustering of samples at 75% Bray-Curtis dissimilarity values identified eight urogenital (**Supplementary figure 2**) and 11 ocular groups (**Supplementary figure 3**). OTUs that were abundant in the dataset and appeared predominant in the heatmap analysis were manually selected and coded as parameters *Propionibacteriaceae_family_06_UGT, Corynebacterium_genus_07_UGT, Aerococcus_genus_03_UGT, Chlamydia_genus_02_UGT, Enterobacteriaceae_family_01_UGT* and *Chlamydia_genus_02_OC*. Clusters were coded as CST_UGT_75 and CST_OC_75. Finally, if a sample had a microbiome that was dominated (>75%) by a single OTU, those microbiomes were coded into the parameter *Monolithic_UGT/OC_microbiome*.

Host genetics. The presence or absence of each allele detected in the study was coded in the appropriate allele parameter: *DAb*10, DAb*15, DAb*21, DAb*30, DAb*31, DAb*32, DAb*33, DAb*34, DAb*35, DAb*36, DBb*01, DBb*02, DBb*03, DBb*05*. In addition, a composite of the complete DAb or DBb allele complement for each animal was coded in the parameter *DAb_profile_type* and *DBb_profile_type*.

References

1. Quigley, B., Ong, V., Hanger, J. & Timms, P. Molecular dynamics and mode of transmission of koala retrovirus as it invades and spreads through a wild Queensland koala population. *J. Virol.* **92,** e01871-17 (2018).

2. Devereaux, L. N., Polkinghorne, A., Meijer, A. & Timms, P. Molecular evidence for novel chlamydial infections in the koala (*Phascolarctos cinereus*). *Syst. Appl. Microbiol.* **26,** 245–253 (2003).

3. Marsh, J., Kollipara, A., Timms, P. & Polkinghorne, A. Novel molecular markers of *Chlamydia pecorum* genetic diversity in the koala (*Phascolarctos cinereus*). *BMC Microbiol.* **11,** 77 (2011).

4. Wan, C. *et al.* Using quantitative polymerase chain reaction to correlate *Chlamydia pecorum* infectious load with ocular, urinary and reproductive tract disease in the koala (*Phascolarctos cinereus*). *Aust. Vet. J.* **89,** 409–412 (2011).

5. Vidgen, M. E., Hanger, J. & Timms, P. Microbiota composition of the koala (*Phascolarctos cinereus*) ocular and urogenital sites, and their association with *Chlamydia* infection and disease. *Sci. Rep.* **7,** 5239 (2017).

6. Oksanen, J. *et al. vegan: Community Ecology Package*. (2017).

Supplementary Table 1. Extended description of factors considered in the structural equation models

**Major category**	**Parameter name**	**Description of parameter**
Physical characteristics	Sex	Female/Male/Intersex
	Age	1-12 years, rounded to whole year
	Body_condition	Vet assessed 3 (poor) to 9 (great) at sampling time
	Weight	1-9 kg
Environmental characteristics	Time of sampling	
	4Season	Spring/summer/autumn/winter time of sampling
	Breeding_season	Breeding season (Sept-Dec) or not at time of sampling
	Breeding_season_longer	Breeding season (Jul-Jan) or not at time of sampling
	Location_simple	1 of 5 regions within study site
Infection urogenital	Infection status	
	Cp_UGT_qPCR_result2	*C. pecorum* qPCR result: not detectable (negative), detectable (positive)
	Cp_UGT_qPCR_result3	*C. pecorum* qPCR result: not detectable (negative), detectable not quanitifiable (low positive), detectable and quantifiable (high positive)
	Infection load	
	Cp_UGT_qPCR_load	*C. pecorum* qPCR result: copies/ul detected
	Cp_UGT_qPCR_load_log	*C. pecorum* qPCR result: log transformed copies/ul detected
	UGT_ompA_type	Genotype of *C. pecorum* based on ompA sequence
Infection ocular	Infection status	
	Cp_OC_qPCR_result2	*C. pecorum* qPCR result: not detectable (negative), detectable (positive)
	Cp_OC_qPCR_result3	*C. pecorum* qPCR result: not detectable (negative), detectable not quanitifiable (low positive), detectable and quantifiable (high positive)
	Infection load	
	Cp_OC_qPCR_load	*C. pecorum* qPCR result: copies/ul detected
	Cp_OC_qPCR_load_log	*C. pecorum* qPCR result: log transformed copies/ul detected
	OC_ompA_type	Genotype of *C. pecorum* based on ompA sequence
KoRV status	KoRV_B	KoRV-B PCR result: not detectable (negative), detectable (positive)
	KoRV_B_epi_subtype	1 of 21 envelope sequence subtypes detected in population
Urogenital microbiome	Phylum-level proportions (comparison by healthy and urogenital disease with independent samples t-test)	
	UGT_Actinobacteria	Proportion of microbiome comprised of this phylum, t(202) = 3.064, p = 0.002
	UGT_Bacteroidetes	Proportion of microbiome comprised of this phylum, t(74) = -2.231, p = 0.029
	UGT_Chlamydiae	Proportion of microbiome comprised of this phylum, t(69) = -2.298, p = 0.025
	UGT_Firmicutes	Proportion of microbiome comprised of this phylum, t(83) = -2.405, p = 0.018
	UGT_Proteobacteria	Proportion of microbiome comprised of this phylum, t(152) = 2.383, p = 0.018

	Genus-level proportions (significantly different genera between health and urogenital disease)	
	UGT_Aerococcus	Proportion of microbiome comprised of this genus, t(70) = -3.061, p = 0.004
	UGT_Chlamydia	Proportion of microbiome comprised of this genus, t(69) = -2.299, p = 0.025
	UGT_Corynebacterium	Proportion of microbiome comprised of this genus, t(187) = 3.364, p = 0.001
	UGT_Facklamia	Proportion of microbiome comprised of this genus, t(169) = 2.644, p = 0.009
	OTU-level proportions (choosen by manual evaluation of OTU-level heatmap)	
	Propionibacteriaceae_family_06_UGT	Proportion of microbiome comprised of this operational taxonomic unit, t(202) = 1.112, p = 0.267
	Corynebacterium_genus_07_UGT	Proportion of microbiome comprised of this operational taxonomic unit, t(187) = 3.364, p = 0.001
	Aerococcus_genus_03_UGT	Proportion of microbiome comprised of this operational taxonomic unit, t(70) = -3.021, p = 0.004
	Chlamydia_genus_02_UGT	Proportion of microbiome comprised of this operational taxonomic unit, t(69) = -2.299, p = 0.025
	Enterobacteriaceae_family_01_UGT	Proportion of microbiome comprised of this operational taxonomic unit, t(164) = 1.872, p = 0.063
	CST_UGT_75	Community state type (1 of 10) determinated at OTU level
	Monolithic_UGT_microbiome	Yes if 1 OTU comprised >75% of the microbiome
Ocular microbiome	Phylum-level proportions (comparison by healthy and ocular disease samples with independent samples t-test)	
	OC_Actinobacteria	Proportion of microbiome comprised of this phylum, t(109) = 2.191, p = 0.031
	OC_Bacteroidetes	Proportion of microbiome comprised of this phylum, t(109) = 2.176, p = 0.032
	OC_Chlamydiae	Proportion of microbiome comprised of this phylum, t(9) = -2.653, p = 0.026
	OC_Firmicutes	Proportion of microbiome comprised of this phylum, t(9) = -0.385, p = 0.709
	OC_Proteobacteria	Proportion of microbiome comprised of this phylum, t(109) = 2.905, p = 0.004
	Genus-level proportions (significantly different genera between health and ocular disease)	
	OC_Chlamydia	Proportion of microbiome comprised of this genus, t(9) = -2.655, p = 0.026
	OC_Flavobacterium	Proportion of microbiome comprised of this genus, t(51) = 6.445, p < 0.001
	OTU-level proportions (choosen by manual evaluation of OTU-level heatmap)	
	Chlamydia_genus_02_OC	Proportion of microbiome comprised of this operational taxonomic unit, t(9) = -2.657, p = 0.026
	CST_OC_75	Community state type (1 of 11) determinated at OTU level
	Monolithic_OC_microbiome	Yes if 1 OTU comprised >75% of the microbiome
Host genetics	DAb*10	MHC class II gene DAb, allele 10, present or absent from host genome
	DAb*15	MHC class II gene DAb, allele 15, present or absent from host genome
	DAb*19	MHC class II gene DAb, allele 19, present or absent from host genome
	DAb*21	MHC class II gene DAb, allele 21, present or absent from host genome
	DAb*30	MHC class II gene DAb, allele 30, present or absent from host genome
	DAb*31	MHC class II gene DAb, allele 31, present or absent from host genome
	DAb*32	MHC class II gene DAb, allele 32, present or absent from host genome
	DAb*33	MHC class II gene DAb, allele 33, present or absent from host genome
	DAb*34	MHC class II gene DAb, allele 34, present or absent from host genome
	DAb*35	MHC class II gene DAb, allele 35, present or absent from host genome
	DAb*36	MHC class II gene DAb, allele 36, present or absent from host genome
	DBb*01	MHC class II gene DBb, allele 01, present or absent from host genome
	DBb*02	MHC class II gene DBb, allele 02, present or absent from host genome
	DBb*03	MHC class II gene DBb, allele 03, present or absent from host genome
	DBb*05	MHC class II gene DBb, allele 05, present or absent from host genome
	DAb_profile_type	Combined profiles of DAb gene presence in host genome
	DBb_profile_types	Combined profiles of DBb gene presence in host genome
Disease categories	Any_Cp_Clinical	Vet assessed chlamydial disease at any body site
	Vet_exam_Cp_UGT_urinary_renal	Vet assessed chlamydial disease at reproductive and/or urinary/renal site
	Vet_exam_Cp_ocular	Vet assessed chlamydial disease at ocular site

Supplementary Table 2 – Structural equation modeling statistics for systematic model evaluation

Dataset	Model description	Model name	P-value (Bollen-Stine) (>0.05)	CFI (>0.9)	RMSEA (<0.05)	# free parameters (K)	Cp R^2	AICc (smaller)	Log likelihood (H0/LL) (larger)	
	Focus on UGT parameters									
204	Phylum micro, breeding season, result2, log load, ompA, UGT/UR Cp disease	AA	1.000	0.995	0.017	20	0.419	4516.37	-2236.54	
204	Phylum micro, breeding season, result2, log load, ompA, any Cp disease	AB	1.000	0.999	0.006	20	0.369	4544.14	-2250.43	worse than AA
204	Phylum micro, X4season, result2, log load, ompA, any Cp disease	AC	1.000	0.996	0.014	20	0.365	4878.01	-2417.36	worse than AA
204	Phylum micro, X4Season, result2, log load, ompA, UGT/UR Cp disease	AD	1.000	0.994	0.020	20	0.415	4850.41	-2403.56	worse than AA
204	Phylum micro, breeding season, result3, log load, ompA, UGT/UR Cp disease	AE	1.000	0.994	0.020	20	0.438	4484.76	-2220.74	better than AA
204	Phylum micro, breeding season, result3, load, ompA, UGT/UR Cp disease	AF	1.000	0.993	0.021	20	0.419	8814.38	-4385.55	worse than AE
204	Phylum micro, breeding season, result3, -log load, ompA, UGT/UR Cp disease	AG	1.000	0.991	0.024	19	0.407	4527.58	-2243.33	worse than AE
204	Phylum micro, breeding season, result3, log load, -ompA, UGT/UR Cp disease	AH	1.000	0.989	0.027	19	0.422	4004.56	-1981.82	better than AE
204	Phylum micro, breeding season, result3, log load, -ompA, move weight to body condition	AI	1.000	0.929	0.054	19	0.378	4016.21	-1987.65	worse than AH
204	Sig Genus micro, breeding season, result3, log load, -ompA, UGT/UR Cp disease	AJ	1.000	0.978	0.039	18	0.416	4324.54	-2142.99	worse than AH
204	OTU micro, breeding season, result3, log load, -ompA, UGT/UR Cp disease	AK	1.000	0.984	0.032	19	0.418	4604.88	-2281.98	worse than AH
204	Phylum micro, +CST, breeding season, result3, log load, -ompA, UGT/UR Cp disease	AL	1.000	0.987	0.028	20	0.424	4562.63	-2259.67	worse than AH
204	Phylum micro, +Monolithic, breeding season, result3, log load, -ompA, UGT/UR Cp diseas	AM	1.000	0.993	0.021	20	0.429	4033.35	-1995.03	worse than AH
204	Phylum micro, breeding season, result3, log load, -ompA, co-vary body condition, UGT/UI	AN	1.000	0.928	0.058	20	0.354	4040.99	-1999.04	worse than AH
204	Phylum micro, breeding season, result3, log load, -ompA, -KoRV epi subtype, UGT/UR Cp	AO	1.000	0.980	0.033	18	0.422	2635.17	-1298.31	better than AH
204	Phylum micro, breeding season, result3, -log load, -ompA, -KoRV epi subtype, UGT/UR Cp	AP	1.000	0.974	0.039	17	0.383	2675.98	-1319.98	worse than AO
204	Phylum micro, breeding season, result2, log load, -ompA, -KoRV epi subtype, UGT/UR Cp	AQ	1.000	0.983	0.030	18	0.409	2712.53	-1336.99	worse than AO
204	Phylum micro, breeding season, result3, log load, -ompA, -KoRV epi subtype, UGT/UR Cp	AR	1.000	0.984	0.028	19	0.429	2579.44	-1269.26	better than AO
204	Phylum micro, breeding season, result3, log load, -ompA, -KoRV epi subtype, UGT/UR Cp	AS	1.000	0.965	0.041	20	0.43	2505.63	-1231.17	better than AR
204	Phylum micro, breeding season, result3, log load, -ompA, -KoRV epi subtype, UGT/UR Cp	AT	1.000	0.957	0.048	19	0.426	2594.07	-1276.58	worse than AS
204	Phylum micro, breeding season (co), result3, log load, -ompA, -KoRV epi subtype, UGT/UI	AU	1.000	0.910	0.058	20	0.363	2615.62	-1286.36	worse than AS
204	Phylum micro, breeding season longer, result3, log load, -ompA, -KoRV epi subtype, UGT,	AW	1.000	0.966	0.041	20	0.428	2507.56	-1232.13	worse than AS
	Best for UGT disease - model AS									
	Focus on OC parameters									
111	Phylum micro, breeding season, result2, log load, ompA, OC disease	BA	1.000	0.943	0.063	20	0.477	1805.99	-879.71	
111	Phylum micro, X4Season, result2, log load, ompA, OC disease	BB	1.000	0.947	0.061	20	0.489	1986.35	-969.89	worse than BA
111	Phylum micro, breeding season, result3, log load, ompA, OC disease	BC	1.000	0.942	0.064	20	0.475	1745.24	-849.33	better than BA
111	Phylum micro, breeding season, result3, log load, ompA, -KoRV epi subtype, OC disease	BD	1.000	0.932	0.067	19	0.474	948.20	-452.20	better than BC
111	Phylum micro, breeding season, result3, load, ompA, -KoRV epi subtype, OC disease	BE	1.000	0.951	0.057	19	0.484	1933.22	-944.72	worse than BD
111	Phylum micro, breeding season, result3, log load, -ompA, -KoRV epi subtype, OC disease	BF	1.000	0.920	0.075	18	0.453	929.95	-444.45	better than BE
111	Sig Genus micro, breeding season, result3, log load, -ompA, -KoRV epi subtype, OC diseas	BG	1.000	0.914	0.088	15	0.419	1185.63	-576.22	worse than BF
111	OTU micro, breeding season, result3, log load, -ompA, -KoRV epi subtype, OC disease	BH	0.990	0.944	0.074	14	0.408	1674.11	-821.72	worse than BF
111	Phylum micro, +CST, breeding season, result3, log load, -ompA, -KoRV epi subtype, OC di	BI	1.000	0.885	0.088	19	0.453	1384.17	-670.19	worse than BF
111	Phylum micro, +Monolithic, breeding season, result3, log load, -ompA, -KoRV epi subtype	BJ	1.000	0.926	0.069	19	0.459	823.80	-390.00	better than BF
111	Phylum micro, +Monolithic, breeding season, result2, log load, -ompA, -KoRV epi subtype	BK	1.000	0.930	0.067	19	0.455	807.05	-381.63	better than BJ
111	Phylum micro, +Monolithic, breeding season, result2, -log load, -ompA, -KoRV epi subtyp	BL	1.000	0.940	0.064	18	0.451	928.33	-443.64	worse than BK
111	Phylum micro, +Monolithic, breeding season, result2, log load, ompA, -KoRV epi subtype	BM	1.000	0.936	0.062	20	0.478	891.00	-422.21	worse than BK
111	Phylum micro, +Monolithic, breeding season, result2, log load, ompA, -KoRV epi subtype	BN	1.000	0.936	0.061	20	0.459	917.74	-435.58	worse than BK
111	Phylum micro, +Monolithic, breeding season, result2, log load, ompA, -KoRV epi subtype	BO	1.000	0.933	0.061	21	0.460	928.12	-439.34	worse than BK
111	Phylum micro, +Monolithic, breeding season, result2, log load, ompA, -KoRV epi subtype	BP	1.000	0.923	0.068	20	0.459	844.43	-389.93	worse than BK
111	Phylum micro, +Monolithic, breeding season longer, result2, log load, -ompA, -KoRV epi s	BQ	1.000	0.961	0.048	19	0.451	838.09	-397.15	worse than BK
	Best for OC disease - model BK									

	Focus on UGT parameters, add in host genetics									
57	Model AS	CA	1.000	0.989	0.026	20	0.613	692.52	-318.41	
57	Model AS, +DAb profile type	CB	1.000	0.957	0.051	21	0.613	1056.08	-498.04	worse than CA
57	Model AS, +DBb profile type	CC	1.000	1.000	0.000	21	0.626	961.58	-450.79	worse than CA
57	Model AS, +DAb10	CD	1.000	0.996	0.014	21	0.617	752.34	-346.17	worse than CA
57	Model AS, +DAb15	CE	1.000	0.964	0.047	21	0.616	754.19	-347.10	worse than CA
57	Model AS, +DAb19	CF	1.000	0.958	0.051	21	0.618	763.75	-351.87	worse than CA
57	Model AS, +DAb21	CG	1.000	0.988	0.028	21	0.649	644.70	-292.35	better than CA
57	Model AS, +DAb30	CH	1.000	0.954	0.053	21	0.622	733.27	-336.63	worse than CA
57	Model AS, +DAb31	CI	1.000	0.981	0.033	21	0.613	700.34	-320.17	worse than CA
57	Model AS, +DAb32	CJ	1.000	0.937	0.063	21	0.638	653.31	-296.66	better than CA
57	Model AS, +DAb33	CK	1.000	1.000	0.000	21	0.615	630.03	-285.01	better than CA
57	Model AS, +DAb34	CL	1.000	0.986	0.029	21	0.615	638.93	-289.46	better than CA
57	Model AS, +DAb35	CM	1.000	0.972	0.041	21	0.613	637.80	-288.90	better than CA
57	Model AS, +DAb36	CN	1.000	0.989	0.025	21	0.615	655.80	-297.90	better than CA
57	Model AS, +DBb01	CO	1.000	0.979	0.350	21	0.613	771.40	-355.70	worse than CA
57	Model AS, +DBb02	CP	1.000	0.992	0.021	21	0.617	754.46	-347.23	worse than CA
57	Model AS, +DBb03	CQ	1.000	0.988	0.027	21	0.628	760.96	-350.48	worse than CA
57	Model AS, +DBb05	CR	1.000	1.000	0.000	21	0.613	771.82	-355.91	worse than CA
57	Model AS, +DAb21, +DAb32, +DAb33, +DAb34, +DAb35, +DAb36	CS	1.000	0.915	0.065	26	0.675	315.59	-115.07	better than CA
57	Model CS, drop UGT microbiome parameters	CT	1.000	0.913	0.075	21	0.631	897.11	-418.56	worse than CS
57	Model CS, drop physical characteristics and location parameters	CU	1.000	0.768	0.118	19	0.553	fail	fail	fail model fit
57	Model CS, drop chlamydia infection parameters	CV	1.000	0.929	0.059	24	0.606	236.25	-80.92	better than CS
57	Model CS, drop ocular disease/infection parameters	CW	1.000	0.964	0.043	24	0.665	292.10	-108.85	better than CS
	Best model of urogenital disease and host genetics - model CV									
	Focus on OC parameters, add in host genetics									
57	Model BK	DA	1.000	0.925	0.077	19	0.633	500.70	-224.55	
57	Model BK, +DAb profile type	DB	1.000	0.911	0.081	20	0.633	826.18	-403.25	worse than DA
57	Model BK, +DBb profile type	DC	1.000	0.940	0.065	20	0.633	765.38	-354.85	worse than DA
57	Model BK, +DAb10	DD	1.000	0.922	0.075	20	0.647	560.77	-252.54	worse than DA
57	Model BK, +DAb15	DE	1.000	0.908	0.083	20	0.664	557.11	-250.71	worse than DA
57	Model BK, +DAb19	DF	1.000	0.924	0.074	20	0.635	570.27	-257.29	worse than DA
57	Model BK, +DAb21	DG	1.000	0.936	0.067	20	0.636	458.39	-201.35	better than DA
57	Model BK, +DAb30	DH	1.000	0.902	0.086	20	0.649	544.30	-244.30	worse than DA
57	Model BK, +DAb31	DI	1.000	0.918	0.077	20	0.639	506.48	-225.39	worse than DA
57	Model BK, +DAb32	DJ	1.000	0.902	0.085	20	0.633	466.48	-205.39	better than DA
57	Model BK, +DAb33	DK	1.000	0.934	0.068	20	0.634	461.96	-203.13	better than DA
57	Model BK, +DAb34	DL	1.000	0.927	0.072	20	0.635	433.22	-188.76	better than DA
57	Model BK, +DAb35	DM	1.000	0.903	0.085	20	0.640	460.19	-202.25	better than DA
57	Model BK, +DAb36	DN	1.000	0.931	0.070	20	0.635	467.73	-206.02	better than DA
57	Model BK, +DBb01	DO	1.000	0.915	0.079	20	0.637	566.85	-255.58	worse than DA
57	Model BK, +DBb02	DP	1.000	0.938	0.066	20	0.635	564.62	-254.46	worse than DA
57	Model BK, +DBb03	DQ	1.000	0.930	0.071	20	0.635	575.75	-260.03	worse than DA
57	Model BK, +DBb05	DR	1.000	0.915	0.079	20	0.633	580.27	-262.29	worse than DA
57	Model BK, +DAb21, +DAb32, +DAb33, +DAb34, +DAb35, +DAb36	DS	1.000	0.909	0.069	25	0.649	168.35	-44.29	better than DA
57	Model DS, drop UGT microbiome parameters	DT	1.000	0.904	0.078	20	0.519	695.55	-319.93	worse than DS
57	Model DS, drop monolithic microbiome parameter	DU	1.000	0.920	0.066	24	0.641	231.31	-78.45	worse than DS
57	Model DS, drop physical characteristics and location parameters	DV	1.000	0.818	0.120	18	0.633	fail	fail	fail to model fit
57	Model DS, drop chlamydia infection parameters	DW	1.000	0.950	0.053	23	0.644	201.53	-66.10	worse than DS
	Best model of ocular disease and host genetics - model DS									

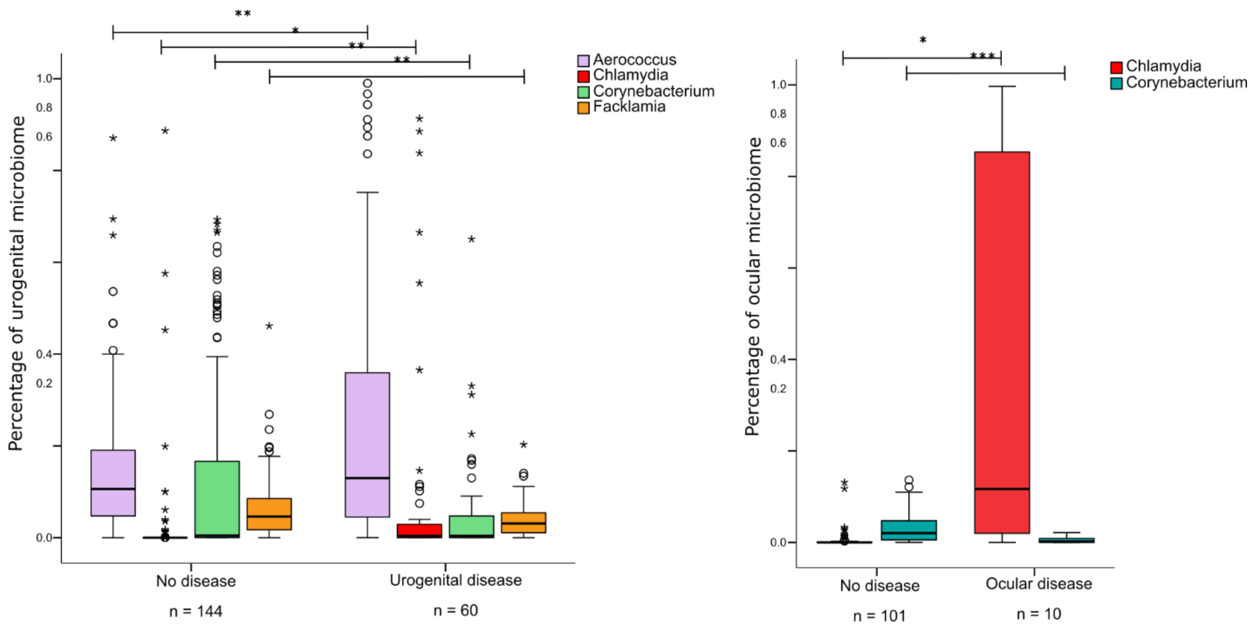

Supplementary figure 1. Genera with significant differences between clinically diseased and healthy koalas at (A) urogenital and (B) ocular sites. Significant differences are represented by * ($p < 0.05$), ** ($p < 0.01$) and *** ($p < 0.001$).

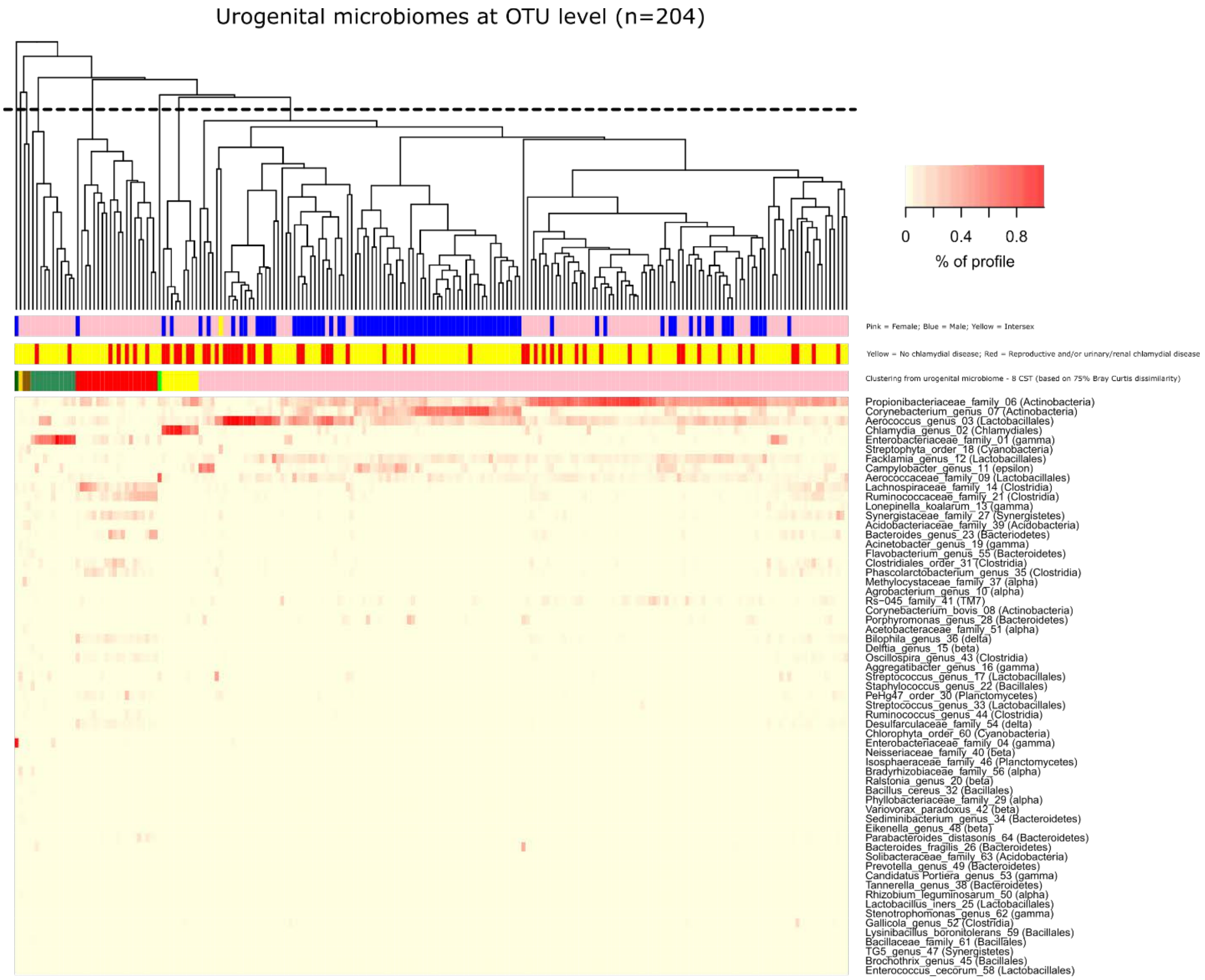

Supplementary figure 2. Urogenital microbiomes at the OTU level. OTU that comprised at least 10% of at least one microbiome (63 OTU in total) were hierarchically clustered (with average linkage) based on Bray-Curtis dissimilarity values. Clusters were determined by grouping samples at 75% Bray-Curtis dissimilarity values, generating 10 urogenital clusters (indicated by dashed line). Koala sex and disease status, along with clusters, are indicated in coloured bars above the heatmap.

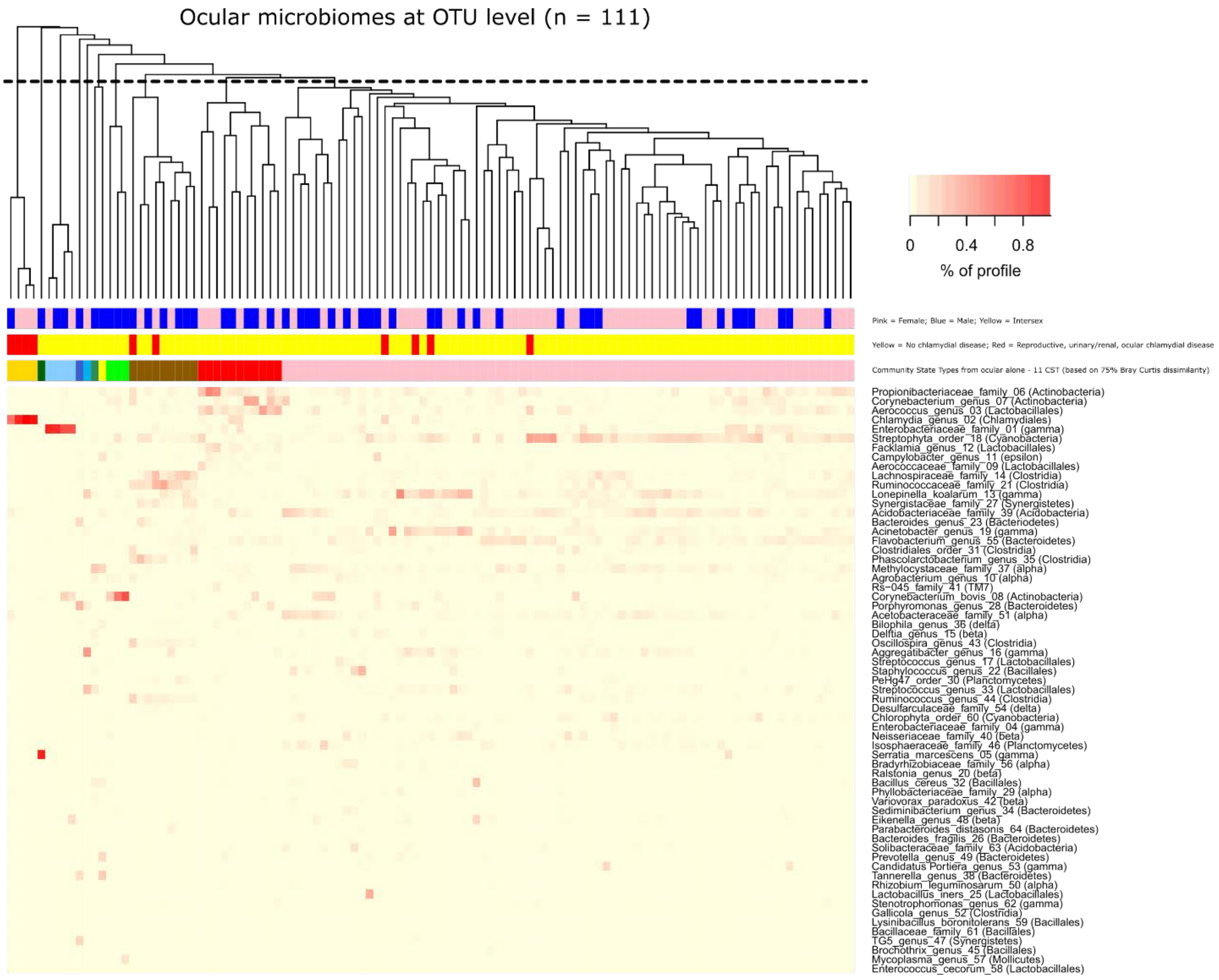

Supplementary figure 3. Ocular microbiomes at the OTU level. OTU that comprised at least 10% of at least one microbiome (63 OTU in total) were hierarchically clustered (with average linkage) based on Bray-Curtis dissimilarity values. Clusters were determined by grouping samples at 75% Bray-Curtis dissimilarity values, generating 11 ocular clusters (indicated by a dashed line). Koala sex and disease status, along with clusters, are indicated in coloured bars above the heatmap.

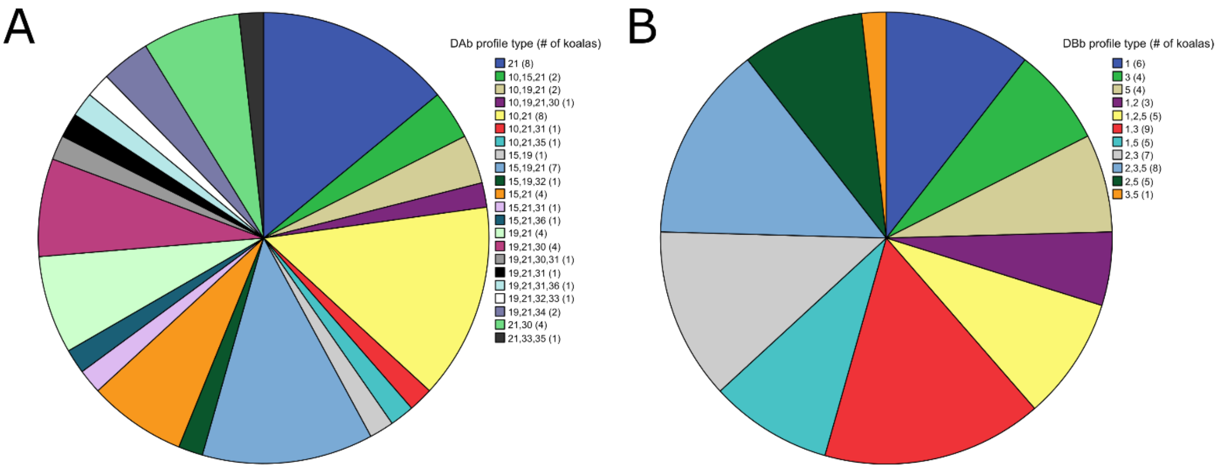

C

Allele	# koalas that have	% of 57 koalas
DAb*10	15	26
DAb*15	17	30
DAb*19	26	46
**DAb*21**	55	96
DAb*30	10	18
DAb*31	5	9
**DAb*32**	2	4
**DAb*33**	2	4
**DAb*34**	2	4
**DAb*35**	2	4
**DAb*36**	2	4
DBb*01	28	49
DBb*02	28	49
DBb*03	29	51
DBb*05	28	49

Supplementary figure 4. Major Histocompatibility Complex (MHC) class II gene allele results from koalas (n = 57). Distribution of (A) DAb allele types and (B) DBb allele types observed in the population. (C) Summary of the individual allele types observed.

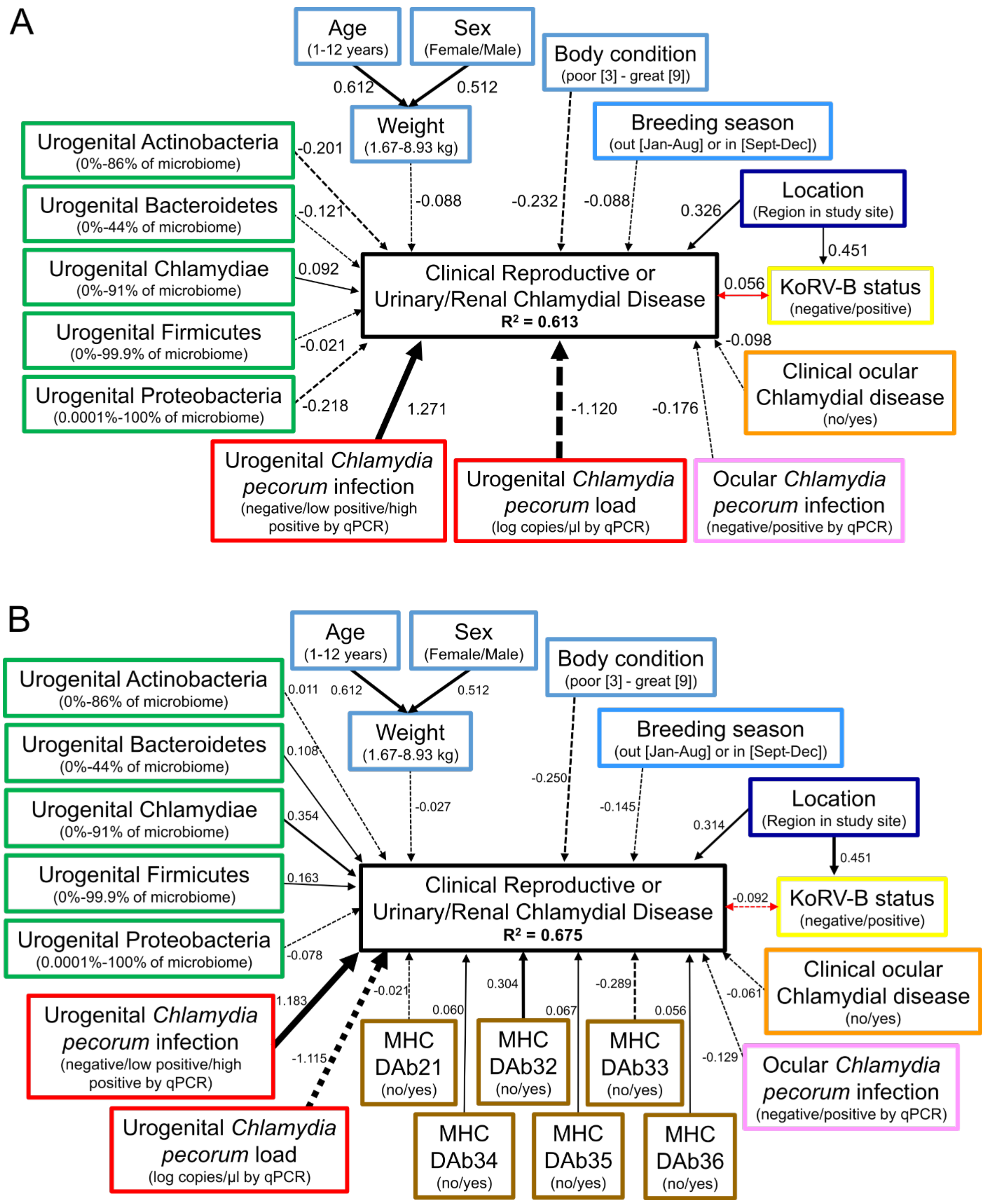

Supplementary figure 5. Preliminary model of clinical urogenital disease with MHC class II gene data added. (A) The original urogenital model rerun with the 57 koala data alone and (B) the same data with the MHC class II data added. The most accurate evaluation of how the MHC data improved prediction should be made to (A) and not Figure 1A.

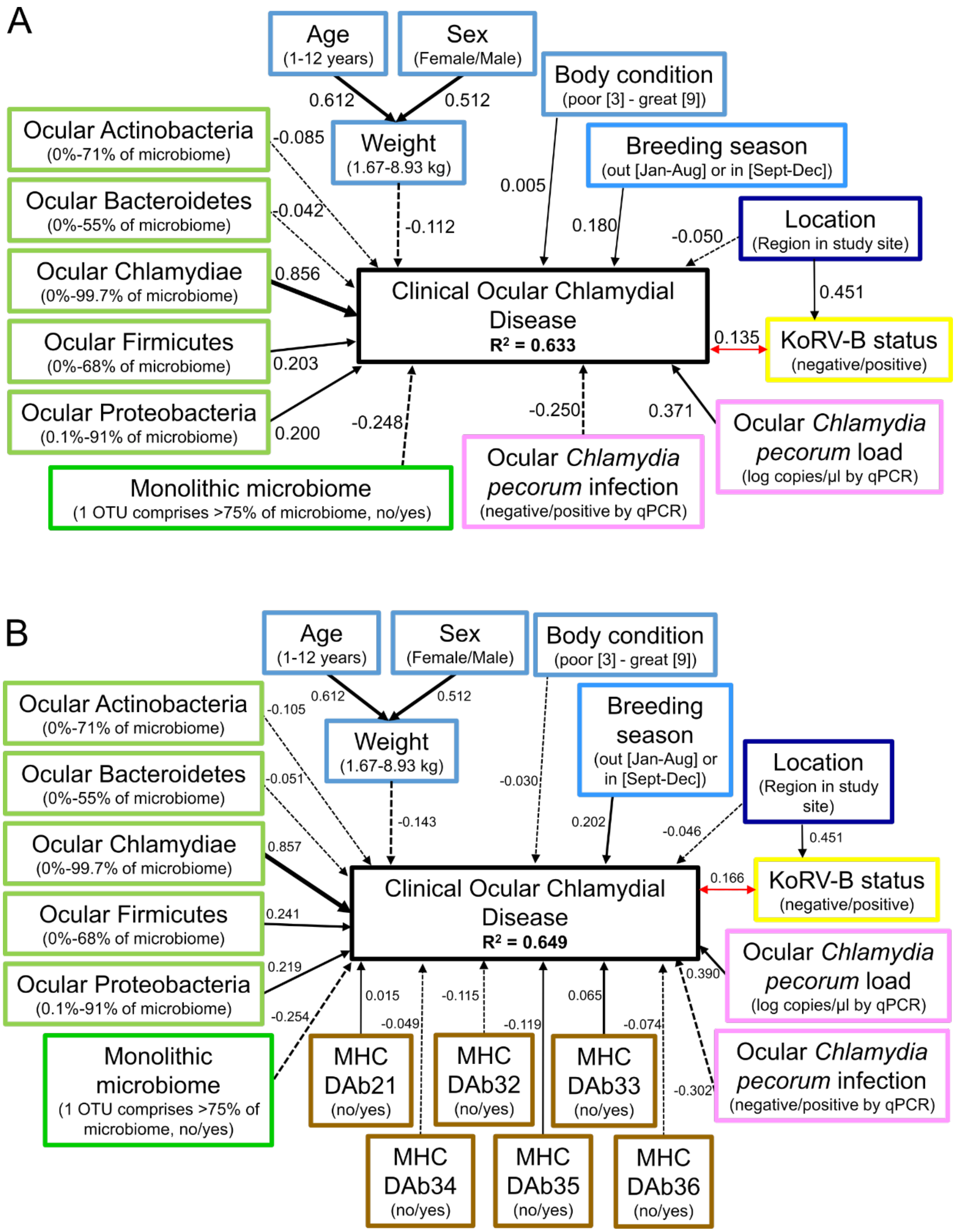

Supplementary figure 6. Preliminary model of clinical ocular disease with MHC class II gene data added. (A) The original ocular model rerun with the 57 koala data alone and (B) the same data with the MHC class II data added. The most accurate evaluation of how the MHC data improved prediction should be made to (A) and not Figure 1C.

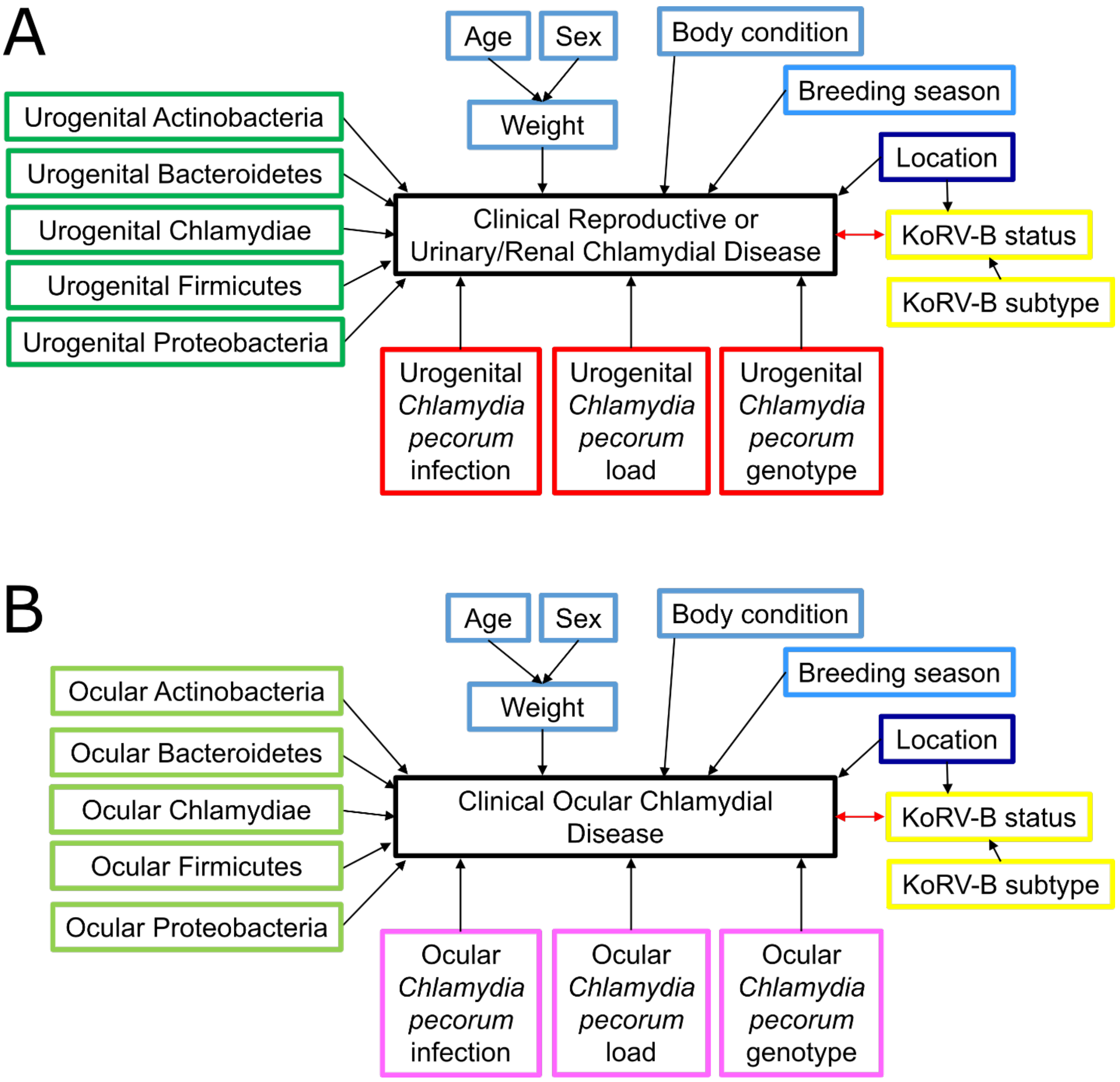

Supplementary figure 7. Starting models for urogenital (A) and ocular (B) structural equation modeling.
